# The ENDORSE study: Research into environmental determinants of obesity related behaviors in Rotterdam schoolchildren

**DOI:** 10.1186/1471-2458-8-142

**Published:** 2008-04-28

**Authors:** Klazine van der Horst, Anke Oenema, Petra van de Looij-Jansen, Johannes Brug

**Affiliations:** 1 Department of Public Health, Erasmus University Medical Center, Rotterdam, The Netherlands; 2 Municipal Health Service for Rotterdam area, Rotterdam, The Netherlands; 3 EMGO Institute, VU University Medical Center, Amsterdam, the Netherlands

## Abstract

**Background:**

Children and adolescents are important target groups for prevention of overweight and obesity as overweight is often developed early in life and tracks into adulthood. Research into behaviors related to overweight (energy balance-related behaviors) and the personal and environmental determinants of these behaviors is fundamental to inform prevention interventions. In the Netherlands and in other countries systematic research into environmental determinants of energy balance related behaviors in younger adolescents is largely lacking. This protocol paper describes the design, the components and the methods of the ENDORSE study (Environmental Determinants of Obesity in Rotterdam SchoolchildrEn), that aims to identify important individual and environmental determinants of behaviors related to overweight and obesity and the interactions between these determinants among adolescents.

**Methods:**

The ENDORSE study is a longitudinal study with a two-year follow-up of a cohort of adolescents aged 12–15 years. Data will be collected at baseline (2005/2006) and at two years follow-up (2007/2008). Outcome measures are body mass index (BMI), waist circumference, time spent in physical activity and sedentary behaviors, and soft drink, snack and breakfast consumption. The ENDORSE study consists of two phases, first employing qualitative research methods to inform the development of a theoretical framework to examine important energy balance related behaviors and their determinants, and to inform questionnaire development. Subsequently, the hypothetical relationships between behavioral determinants, energy balance related behaviors and BMI will be tested in a quantitative study combining school-based surveys and measurements of anthropometrical characteristics at baseline and two-year follow-up.

**Discussion:**

The ENDORSE project is a comprehensive longitudinal study that enables investigation of specific environmental and individual determinants of overweight and obesity among younger adolescents. The project will result in specific recommendations for obesity prevention interventions among younger adolescents.

## Background

Adolescent overweight and obesity are important public health concerns in the Netherlands as well as in other western countries, due to the increasing proportion of adolescents classified as overweight or obese [1]. Children and adolescents are an important target group for intervention activities aimed at the prevention of overweight. Overweight and obesity often manifest early in life [2,3] and is associated with an increased the risk of serious diseases during childhood and adolescence [4]. Furthermore, obese children and adolescents are likely to become obese adults, who have an increased risk for various chronic diseases and premature death [5]. Therefore, it is important to develop interventions that prevent children and adolescents from gaining excess weight. Prevention of weight gain can best be achieved by focusing on both sides of the energy balance equation; energy intake (diet) and energy expenditure (physical activity). To be able to develop theory and evidence-based interventions aimed at the prevention of excess weight gain, it is essential to identify which specific energy intake and energy expenditure behaviors contribute most to excess weight gain, and which determinants mediate or predict engagement in such behaviors.

An important development in overweight and obesity prevention research has been the recognition of the environment as a potentially important determining factor for energy balance related behaviors [6]. Currently, there is only limited scientific evidence regarding the influence of environmental determinants on energy balance related behaviors among adolescents [7,8]. Few studies have examined the relative importance of environmental determinants and individual (cognitive) determinants that have been the more traditional focus of behavior change interventions, and there is also lack of empirical evidence regarding the interactions between these determinants [9-11]. Research into environmental determinants is now emerging and more rigorous and well-designed studies are needed to draw stronger inferences for relationships between environmental determinants, energy balance related behaviors and BMI. Such studies are also needed to identify the interactions between determinants of various energy balance related behaviors and the mechanisms underlying the associations between individual and environmental determinants of these behaviors [6,11]. Therefore, a comprehensive study was designed that examines key energy balance related behaviors, the individual and an environmental determinants of these behaviors and that contains objective measures of height, weight and waist circumference. The target population of the study is adolescents aged 12–15 years. The specific aims of the study are: (i) to identify important behaviors related to overweight (energy balance related behaviors), (ii) to examine important individual (cognitive) and environmental determinants for the energy balance related behaviors identified, (iii) to investigate the associations with and the interactions between these determinants and BMI in cross-sectional and prospective analyses with a two year follow-up, and (iv) to formulate objectives to be targeted in interventions aimed at the prevention of overweight in adolescents aged 12–15 years.

The ENDORSE study (Environmental Determinants of Obesity in Rotterdam's SchoolchildrEn) is conducted within the Center for Effective Public Health In the larger Rotterdam area (CEPHIR), an established collaboration between a university research center (Erasmus MC) and the Municipal Health Service Organization in the Rotterdam region. The data collection process takes place in close cooperation with the Municipal Health Service Rotterdam area, using an existing research infrastructure. In this article we describe the design and protocol of the ENDORSE study.

The study comprises of two parts. The first part focused on the identification of the key energy balance related behaviors and the important individual and environmental determinants to examine. Based on this identification, measurement instruments were developed. The second part consists of a combined cross-sectional and longitudinal study utilizing these instruments.

### Pilot work: Identification of risk behaviors and important environmental determinants

This phase of the ENDORSE study involved the development of questionnaires for adolescents and parents, interview forms for school representatives and canteen managers, an audit instrument used to observe the school and the neighborhood around schools, and a list of important census data on neighborhood level. To develop these instruments systematically, important behaviors and determinants were identified in the following steps.

#### Identification of important energy balance related behaviors in youth

The most important energy balance related behaviors were identified to gain insight in the contribution of these behaviors to overweight and obesity in adolescents. Based on a review of relevant reviews of the literature, a preliminary list of specific relevant energy balance related behaviors was compiled. This list contained: watching television, computer use, sports, physical education, transport to school, leisure time activities, soft drink consumption, skipping breakfast, consumption of foods high in fat, fruit and vegetable consumption, portion sizes and dining out. Subsequently national experts on energy balance related behaviors were asked to review this list and suggest additional important behaviors and score the behaviors on the importance and changeability of each behavior. This procedure resulted in the following identification of behaviors to be examined in the present study: active transport to school, leisure time activities, sports, watching television, computer use, soft drink consumption, sweets/cookies/cake/chocolate bar consumption, savory snack consumption and breakfast consumption.

#### Identification of environmental and individual determinants of energy balance related behaviors among youth

In the ENDORSE study the environment was defined as 'anything outside the individual'. The environment can be subdivided by means of distinguishing various environmental factors. A suitable framework for the classification of environmental determinants is the Analysis Grid for Environments Linked to Obesity (ANGELO) [12]. This framework was specifically developed to conceptualize health behavior environments, and enables the identification of potential intervention settings and strategies. According to the ANGELO grid, environmental determinants can be grouped in four environmental types (physical, socio-cultural, economic, and political) and specific environmental levels (micro and macro). To integrate important environmental types and levels in the ENDORSE study; physical, socio-cultural, economic and policy determinants were examined at the micro level (home, school and neighborhood level). Combinations of perceived and objectively measured environmental determinants were used to investigate the interactions between environmental and individual determinants.

Environmental determinants previously shown to be important were identified by conducting two systematic reviews, one with physical activity as the outcome behavior [7] and the other with specific obesity related dietary behaviors as outcome [8]. The results from the reviews were categorized using the ANGELO grid. Convincing evidence of an important role for physical environmental determinants was not found. However, only a limited number of studies assessing physical environmental determinants of energy balance related behaviors were retrieved. Most consistent determinants of physical activity in adolescents were support from significant others, mother's education level, family income and non-vocational school attendance and low neighborhood crime incidence [7]. The most consistent determinants of obesity related dietary behaviors among adolescents were parental and family influences, e.g. parental and sibling intakes, parenting style, family connectedness and parental education [8]. The results of the reviews were used to guide the design of questionnaires and observation forms. Since the evidence from the reviews itself was not sufficient, potential determinants of physical activity and dietary behaviors were also included in the measurement instruments.

The theory of planned behavior (TPB) was used for the selection of potential individual determinants to be included in the study [13]. The TPB postulates that intention to perform a behavior, the determinant most proximal to behavior, is determined by three conceptually independent constructs: attitude, subjective norms and perceived behavioral control. To further explore what specific concepts, beliefs or perceptions would be important for adolescents; focus group interviews with adolescents were held. A focus group interview is conducted among a small group of people who, led by a moderator and following a predetermined interview scheme, discuss several topics related to a specific subject. The aim of the focus groups was to gain insight in the individual and environmental determinants of snacking, soft drink consumption, eating breakfast and physical activity. Three schools participated and teachers were asked to select adolescents who would be able to function in a group discussion (i.e. who were not too shy or too dominant). Five focus groups were conducted with seven to nine adolescents aged 13–15 years old, and a total of 39 adolescents participated. Two of these groups consisted of boys only; two of girls only; and one was a mixed group with boys and girls. Three of the groups were composed of adolescents from cultural and ethnic minorities reflecting the cultural diversity of the residents of Rotterdam. Each interview was tape-recorded and lasted about 45 minutes. The focus groups were transcribed verbatim and from these transcripts, quotes were categorized into the determinants or concepts that they reflected. Many adolescents identified that seeing other people eating or drinking and smelling fast food were factors that influenced their eating and drinking patterns. These factors can be translated as the concept 'external cues'. Assessment of a tendency to respond to external cues was therefore included in the adolescent questionnaire as an individual determinant. Rules at home (e.g. not allowed leaving the house without eating breakfast) or the lack of rules (e.g. allowed to drink as much soft drinks the adolescent wants) were also mentioned by participants. Parental influences already were identified as potential important determinants from the systematic reviews, and based on the results of the focus group interviews, items examining parents' rules or 'parenting practices' were included in the adolescent and parent questionnaires.

## Methods

### Design

The ENDORSE study has a cross-sectional and a prospective two year follow-up component. Data will be collected at baseline (2005/2006) when adolescents aged 12–15 years, and two years later (2007/2008). Outcome measures are body mass index (BMI), waist circumference, physical activity, sedentary behaviors, and soft drink, snack and breakfast consumption. The study is an integral part of the ongoing health surveillance system of the Municipal Health Service in the Rotterdam area (Youth Monitor Rotterdam), in which general health, well being and related factors of youth aged 0–19 years are monitored. The Medical Ethics Committee of Erasmus University Medical Center reviewed the proposal and issued a "declaration of no objection" for the ENDORSE project.

### Recruitment of schools

Schools located in the Rotterdam area that participate in the Youth Monitor Rotterdam (YMR) (N = 56) were invited for participation in the ENDORSE study. A letter and an information sheet explaining the goals and the logistics of the study were sent to school principals. The schools Principals were contacted by a researcher, upon which they could express their interest in participating in the study. If necessary, additional information to make a more informed decision was provided. Subsequently, a random sample of 17 school locations was drawn from the pool of schools that were willing to participate, after stratification of the schools according to the area in the city in which they are located. Stratification was done, to ensure a range of physical and cultural environments. Rotterdam is the second largest city of the Netherlands. It has approximately 600,000 inhabitants of which 46% are of non-Dutch origin [14].

### Recruitment of participants within schools

Five classes in each participating school were randomly selected for participation in the cross-sectional study, which took place in 2005/2006. All adolescents in one class participated in the study, unless they or their parents indicated that they were not willing to participate. The adolescents in the first year classes were also asked to complete the questionnaires at two years follow up. To have sufficient power, we assumed (conservatively) that obesity inducing risk behaviors will be present among at least 40% of adolescents, and we assumed moderate effect sizes of determinants on behaviors. With a significance level of .05 and 80% power, a sample size of approximately 600 students would be sufficient. Since we plan to do separate analyses for girls and boys we aimed at including 1200 students. First year adolescents were over-sampled to 800 as these adolescents will be followed up two years later.

### Procedure

The ENDORSE study follows the logistics of the YMR. The YMR routinely collects data among adolescents in the first and third year of secondary school. The school levels vary from lower vocational training to high school. According to the usual procedure of the YMR, the ENDORSE study was announced through a letter to the parents. This letter explained that the YMR was extended with an extra part, aimed to gain insight in the prevalence and causes of overweight. Parents could keep their child from participating in the study by sending the attached form to the adolescent's teacher (passive consent procedure). Approximately two weeks after the usual YMR questionnaire, the adolescents completed the ENDORSE questionnaire confidentially during a school hour with a teacher and a research assistant present. Within a month after completion of the ENDORSE questionnaire, two trained research assistants measured height, weight, waist circumference and pubertal development according to standardized procedures described in a measurement protocol. The adolescents were asked to come in succession to a private room where they were measured without shoes. After the anthropometrical measurements, the adolescents completed a Tanner scale to assess pubertal development [15]. To guarantee confidentiality the adolescents could put the Tanner scale form in an envelope before handing it over to the research assistant. After these measurements, the adolescents received a Frisbee as a compensation for their participation and were requested to give an envelope with a questionnaire to their parents. The envelope contained a letter explaining the purpose of the study and the reason why the parents were asked to complete the questionnaire, a pre-addressed and stamped envelope and a card which they could complete to participate in a raffle to win one of five I-pods. Parents were reminded twice to complete and return the questionnaires by means of reminder cards delivered to parents via the adolescents. Parents were not addressed directly, since the YMR procedure did not allow us to have any personal or address details.

Two observers independently conducted audits of the schools, school canteens, schoolyards, and an area of 300 meter radius surrounding the schools. The observations were conducted within three months from the completion of the adolescent questionnaire. A brief interview with canteen managers and school representatives was part of the audit. One of the observers conducted the interviews with school representatives, and the other observer conducted the interviews with the school canteen managers.

Census data (year 2005/2006) from the Center for Research and Statistics (COS), the research center of the municipality of Rotterdam was collected on all neighborhoods of Rotterdam.

In the follow-up data collection the same procedures are used. All measurements (questionnaire, anthropometrics, audits and interviews) are conducted within one week per school. As an incentive for their participation, the adolescents received a key holder.

### Measurements

The ENDORSE questionnaires were developed by using existing validated Dutch questionnaires where possible. If no validated questionnaires were available the ENDORSE questionnaires were informed by questionnaires on related topics that were used in ongoing projects in the Netherlands, and questionnaires used in other countries. Relevant parts of these questionnaires were adapted to tailor the specific behaviors identified. If no relevant and validated questionnaires were available, new questions were developed for the ENDORSE study. The ENDORSE study contained the following measurements: adolescent questionnaire, parent questionnaire, interviews with school representatives and canteen managers, observations of the school environment, census data collection and adolescent body measurements. These are described in detail in the following paragraphs. All determinants measured in the ENDORSE study are listed in Table 1.

**Table 1:** Individual and environmental correlates measured in the ENDORSE study

	**Perceived/self-reorted variables**			**Objectively measured variables**	
	**Adolescent questionnaire**	**Parent questionnaire**	**Interview**	**Observation**	**Census data**

**Individual variables**	Attitude; parental norms; modeling; perceived behavior control; intention; habit; external eating behavior				
**Physical environment**	Home*:	**Home:**		**School:**	**School:**
	Availability of sports facilities, bicycle; soft drinks, breakfast products, snacks/sweets, television set in bedroom.	Availability of bicycles, cars, soft drinks, breakfast products, snacks/sweets		Availability of bicycle shed, food products in the school canteen and vending machines, PA facilities on the school playground; Shops, fast food restaurants & PA facilities in the school neighborhood. Traffic amount and safety Facilities and frequency of public transport	Availability of shops, sports facilities and playgrounds for children > 12 years old; areas of sidewalks, bicycle lanes, roads, grass, plants, water; traffic accidents; criminality, crime reports
	Accessibility of soft drinks, snacks/sweets, breakfast products, television set	Accessibility of soft drinks, snacks/sweets, breakfast products, television set			
	**School:** Amount of traffic; safety for cycling; availability of sidewalks and cycle lanes; availability of a bicycle shed	**Neighborhood**: Amount of traffic; safety for cycling; availability of sidewalks and cycle lanes; safety in neighborhood; attractiveness of neighborhood			**Neighborhood:**Availability of shops, sports facilities and playgrounds for children > 12 years old; areas of sidewalks, bicycle lanes, roads, grass, plants, water; traffic accidents; criminality, crime reports
	**Neighborhood**: Amount of traffic; safety for cycling; availability of sidewalks and cycle lanes; safety and attractiveness of neighborhood; availability of playgrounds, parks, squares, sports clubs				
**Socio-cultural environment**	**Home:** Having family breakfast and dinner; parental allowance; parenting practices; parenting style	**Home:** Having family breakfast and dinner; parental allowance; parenting practices; parental behavior; # persons in household			**Neighborhood**: % residents aged 10–19
**Economic environment**	**Home:** Income; amount of money that can be spent in 1 week	**Home:** Having a paid job; educational level		**School:** Pricing of school canteen products; pricing of products in shops around the school	**Neighborhood**: Residential types; household income; educational level; % unemployment, % living on social security: % rented houses/owner-occupied properties; mean value of houses; % various ethnic groups
**Political environment**			**School**: Food & physical activity policy		

* Home, school, or neighbourhood refers to the specific environmental level of the determinants; e.g. the availability of soft drinks can be assessed in the home environment of the adolescent or at the school level.

#### Adolescent questionnaire

Physical activity and sedentary behaviors were assessed with an adapted version of the Activity Questionnaire for Adolescents & Adults (AQuAA) [Chin A Paw MJ, Slootmaker SM, Schuit AJ, van Zuidam M, Van Mechelen W, Journal of Clinical Epidemiology, Journal of Clinical Epidemiology, unpublished 2008] which is a short questionnaire to assess physical activity at school and during leisure time, active transportation to school and sedentary behaviors in leisure time. The structure of the AQuAA was obtained from the SQUASH-questionnaire [16]. The AQuAA refers to activities in the past week (7-day recall). The test-retest reproducibility was fair to moderate for this questionnaire, with intra-class correlations ranging from 0.46 to 0.59.

Dietary intake was assessed with food frequency questions referring to a general week, and a 24-hour recall question. The questionnaire included TPB items for all behaviors. All the questions on TPB variables were measured on a five-point bipolar scale. Attitude was assessed with two items by asking if the adolescent considered the behavior as good or bad, and as pleasant or unpleasant ('e.g. Regular physical activity is very good (+1) – very bad (-1)'). Subjective norm was assessed with one item, for example 'my parents consider eating breakfast as very good (+2) – very bad (-2)'. Modeling was assessed with two items by asking if the parents and friends perform the behavior (' My friends eat snacks...a lot (+2) – very little (-2)'). Perceived behavioral control was assessed with two items by asking how easy or difficult the behavior is to perform (How easy/difficult is it for you to eat breakfast? Very easy (+2) – very difficult (-2)), and by asking if the decision to perform a behavior is completely under the control of the adolescent (Do you decide by yourself if you eat breakfast? Yes, that is completely my own decision (+2) – no, that is not fully my own decision (-2)). Intention to perform the behavior was assessed with one item asking how certain the adolescent is to perform the behavior in the coming six months (Do you intend to eat breakfast the next six months? Yes, certainly do (+2) – no certainly do not (-2)).

Habit strength of dietary and physical activity behaviors was measured by means of the Self Report Habit Index [17]. This questionnaire assesses three features of habitual behavior: the extent to which a behavior is automatic, the repeated character of the behavior and the sense of identity the behavior reflects. Three items assessed these features, namely: the behavior 'x' is something.... 'I do frequently', 'is something I do automatically' and 'is something that's typically 'me". These items were measured on a five-point scale, ranging from 'I completely agree' (+2) to 'I completely disagree' (-2).

External cues that can influence eating and drinking patterns were questioned with nine items on a four point Likert scale (always (+2) – never (-2)), for example 'I get hungry when I see snacks or candy' or 'When I walk past a fast-food restaurant, I feel like buying something. These questions were based on the external eating behavior questions from the Dutch Eating Behavior Questionnaire [18] and adapted to address the topics adolescents mentioned in the focus group interviews.

In the adolescent questionnaire the following perceived environmental determinants were assessed: availability and accessibility of facilities for physical activity and food, school factors, neighborhood factors, parenting factors and economic factors. Demographic factors (gender, age, ethnicity) were available for each adolescent through the YMR questionnaire.

The adolescent questionnaire was pre-tested among ten adolescents by means of cognitive interviewing. Subsequently, the questionnaire was completed twice by 89 schoolchildren (aged 13–14) ten-days apart to assess the test-re-test reliability and other psychometrics of the questionnaire. Items with low reliability were adjusted or deleted from the questionnaire.

#### Parent questionnaire

Parental behavior, family and household environmental determinants were assessed in the parent questionnaire. Parental physical activity and sedentary behaviors were assessed with the adapted version of the AQuAA [Chin A Paw MJ, Slootmaker SM, Schuit AJ, van Zuidam M, Van Mechelen W, Journal of Clinical Epidemiology, unpublished 2008], referring to activities in the past week (7-day recall). Dietary behaviors were assessed with food frequency questions referring to a general week. Neighborhood factors as perceived by the parents, such as safety in neighborhood and attractiveness of neighborhood, parenting practices, parental allowance, availability and accessibility of soft drinks, breakfast products, snacks/sweets, television set and parental self-reported body weight and height, and demographics (gender, educational level, having a paid job) were assessed. One parent completed this questionnaire.

#### Interview questionnaires

To assess school food and physical activity factors, pre-structured interview forms were developed for interviews with the school canteen managers and with a school representative. The interview form for canteen managers contained questions on the availability of food products, opening hours of the canteen, pricing policy, who will receive profits from the foods sold, and canteen policies, for example if there are agreements on the assortment with the catering organization. The form also included information about the soft drink and snack vending machines, e.g. how often the vending machines are filled. The interviews with a school representative were aimed at gaining insight in school policies regarding diet and physical activity. As a basis for the interview form, the 7-item school-wide food practices scale was used [19]. This scale assesses food practices allowed at school with the following items: 'Are students allowed to have food in the classroom', 'Are students allowed to have beverages in the classroom?', 'Are students allowed to have snacks in the hallways?', 'Are students allowed to have beverages in the hallways?', 'Are food or food coupons used as reward or incentive for students?', 'Do you have classroom fundraising that includes food sales?', and 'Do you have school wide fundraising that includes food sales?'. The questionnaire furthermore contained questions about whether or not the school has a formal food or physical activity policy and if yes, to indicate what this policy is. Questions on what health education programs they use in schools and possibilities and promotion activities for the adolescents to be active before, during and after school time, were also included.

### Audit instrument for area observations

An audit instrument was developed to assess the availability and accessibility of foods and physical activity facilities in the schools, in the schoolyards and in the neighborhood around schools. The audit instrument consists of a pre-structured form with five parts: school information, school building, nutrition, physical activity and school environment. As much as possible, the instrument had a 'tick box' answering format and included observation of 'objective' characteristics. When more subjective characteristics such as 'state of maintenance of the school yard', or 'traffic situation around the school' were reported, photographs were taken from pre-defined angles. The audit instrument included also a description of the item to be observed. The neighborhoods around schools that were observed were defined as a radius of 300 meters from the school. This definition was based on the basic assumption that the facilities in the neighborhood around schools should be accessible in a general school lunch break of approximately 30 minutes, and that adolescents use facilities that are close by the school. The audit instrument included maps of the 300-meter radius around the schools, on which the route walked to observe the area could be drawn and the location of green spaces could be indicated. The first part (A) of the audit instrument involved some general school information e.g. the address and number of pupils. Part B involved items on the school building e.g. number of floors, entrance for schoolchildren, availability of an elevator and location and visibility of stairs. Part C involved observation of the school canteens e.g. counting the number of soft-drink and snack vending machines in the canteen, checking the items that are available in these machines, how well they were filled and advertising in the canteen. Part D involved observation of the school physical activity facilities e.g. the bicycle shed, facilities for activity and aesthetics of the schoolyards. Part E involved observation of the neighborhood around schools e.g. the facilities for physical activity (e.g. parks, fields, playing and sports fields) that were visible from the schoolyards and that were present in the neighborhood (300 meters). The component of the neighborhood observation related to dietary intake involved observation of the food retail outlets (baker's shops, snack bars, fast-food chains, supermarkets, kiosks, gas stations, tobacco shops, chemist's shops) that were visible from the schoolyards and that were present in the neighborhood around schools.

The audit instrument first was reviewed by experts on accuracy and completeness of the instrument for its intended purpose. Secondly, the instrument was pilot tested by conducting the observations at two schools and in the corresponding neighborhoods with two observers. Important aspects of the pilot test were the completeness of the forms and feasibility and suitability of using the definition of 300-meter radius for school environment. After the pilot tests at the two schools, the audit forms were adapted if needed. The adapted forms were tested at a third school, by three observers.

### Census data

Census data was utilized to gather additional environmental data regarding the neighborhoods around schools and the neighborhoods in which the children live. The data included area-level household income, educational level, residential types, percentage of residents aged 10–19, percentage unemployment, percentage living on social security, percentage of rented houses and owner-occupied properties, mean value of houses, percentages of various ethnic groups, number of stores, fast food restaurants and the amount and type of green spaces, water, bicycle tracks and foot paths. The census data could be linked to the home environment of the adolescents with information on the ZIP code, which was asked in the adolescent and parent questionnaires. Neighborhoods were defined based on a formal classification from Statistics Netherlands.

### Body measurements

Body height was measured without shoes with a Seca 225 mobile height rod with an accuracy of 0.1 cm. A calibrated electronic digital floor scale (SECA 888 class III) was used to determine body weight of the participant in street clothes, without shoes, with an accuracy of 0.1 kg. Waist circumference was measured using a spring loaded measuring tape (SECA 200) to the nearest 0.1 cm. The waist circumference was measured twice. In case of a difference of more than 1.0 cm between these two measurements, the waist circumference was measured twice again. Adolescents self-reported on their stage of pubertal development using drawings of Tanner stages [15] in the baseline data collection only.

## Discussion

The ENDORSE study is a comprehensive, longitudinal study in which both individual and environmental determinants of selected obesity related behaviors in adolescents are examined. The study has several strengths. It examines both sides of the energy balance equation. It was designed to examine the influence of environmental factors on obesity related behaviors and BMI and objective measures for mapping the environment were included. Moreover, the study includes environmental factors in various settings, including the home, school and neighborhood. The study involves assessments of both individual and environmental determinants, as opposed to many previous studies, that focused on one or the other. The study has a longitudinal design, allowing analyses of prediction rather than cross-sectional associations only. To date, there are very few studies that examine environmental factors of energy balance related behaviors longitudinally. There is an urgent need for such studies, in order to be able to draw stronger inferences for relationships between environmental factors and BMI. However, there were also some limitations in the study protocol. For instance previously validated instruments were not available for all necessary measures. Another limitation is that assessments of adolescents' and parental physical activity and dietary behaviors are self-reported. The definition of environment and neighborhood is also somewhat arbitrary. The scale of environment to be studied needs further conceptual development [20]. A clear definition of 'the neighborhood' is needed in terms of measurement of respondent perceptions and objective measures of the environment. However there is to date little evidence or consensus as to what constitutes a neighborhood. There is poor agreement about which boundary or scale to use, and how this might impact on the association between predictor and outcome variables is unknown. Moreover the boundary to be used might differ for different target groups and different settings (school or home environments) [20].

The ENDORSE study contains rich data examining individual and environmental determinants of energy balance related behaviors among adolescents. With this information the influence of risk behaviors for overweight and relationships with socio-economic status and ethnicity can be investigated. Individual and environmental determinants of obesity-related behaviors among adolescents can be examined as well as the interactions between individual and environmental determinants of obesity inducing behaviors. Therefore, data will be analyzed by means of multi-level regression analyses and structural equation modeling. Eventually, the ENDORSE study will provide objectives and entry points for prevention of overweight interventions in younger adolescents. In 2008 the questionnaires for adolescents and parents, the school policy interview forms and the audit instrument will be made available on the Internet [21].

## Competing interests

The authors declare that they have no competing interests.

## Authors' contributions

KvdH drafted the study protocol with input from all authors. All authors read and approved the final manuscript.

## Pre-publication history

The pre-publication history for this paper can be accessed here:


